# Unmasking the Masked: A Case Report of Atypical Methotrexate Toxicity Leading to Severe Bone Marrow Suppression

**DOI:** 10.7759/cureus.93828

**Published:** 2025-10-04

**Authors:** Dipesh Karki, Roshna Shrestha, Asmi Pandey, Prashant Neupane

**Affiliations:** 1 Internal Medicine, B and C Medical College Teaching Hospital and Research Center, Birtamod, NPL; 2 Internal Medicine, Liverpool Heart and Chest Hospital NHS Foundation Trust, Liverpool, GBR; 3 Vascular Surgery, Manchester Royal Infirmary, Manchester, GBR

**Keywords:** bone marrow suppression, dosing errors, methotrexate, methotrexate toxicity, rheumatoid arthritis

## Abstract

Methotrexate (MTX), a cornerstone in the management of rheumatic diseases, can induce severe adverse effects if not monitored meticulously. This report details a rare case of MTX-induced severe bone marrow suppression in a 65-year-old female, highlighting the critical importance of dosing accuracy, especially in the elderly. The patient, diagnosed with rheumatoid arthritis, experienced an acute episode of toxicity due to a dosing error. This case underscores the necessity for stringent monitoring and patient education to prevent similar incidents.

## Introduction

Methotrexate (MTX), a systemic immunosuppressive agent, is extensively used as a disease-modifying antirheumatic drug (DMARD) in various connective tissue disorders. The dosage used varies from 7.5 mg to 30 mg once weekly [1]. While its efficacy is well-documented, MTX toxicity remains a significant clinical concern, particularly in the context of dosing errors. Besides well-known side effects like bone marrow suppression, it can also cause uncommon skin reactions, including cutaneous ulcers [2].

## Case presentation

History

A 65-year-old woman, a known case of rheumatoid arthritis (RA) for the past four years, presented to our clinic with painful, haemorrhagic ulcers on her lips and the corners of her mouth, a single episode of low-grade fever, and painful swelling in her hands and feet. She also complained of a few episodes of loose stool. There was no significant family or psychosocial history. She denied any history of drug allergies. She was on leflunomide 20 mg daily for RA and etoricoxib for pain relief for bilateral knee osteoarthritis. A month ago, she was prescribed MTX 7.5 mg twice weekly and folic acid 5 mg once weekly for better control of her RA. However, she was unintentionally taking MTX 7.5 mg twice daily instead of twice weekly.

Examination

**Figure 1:**
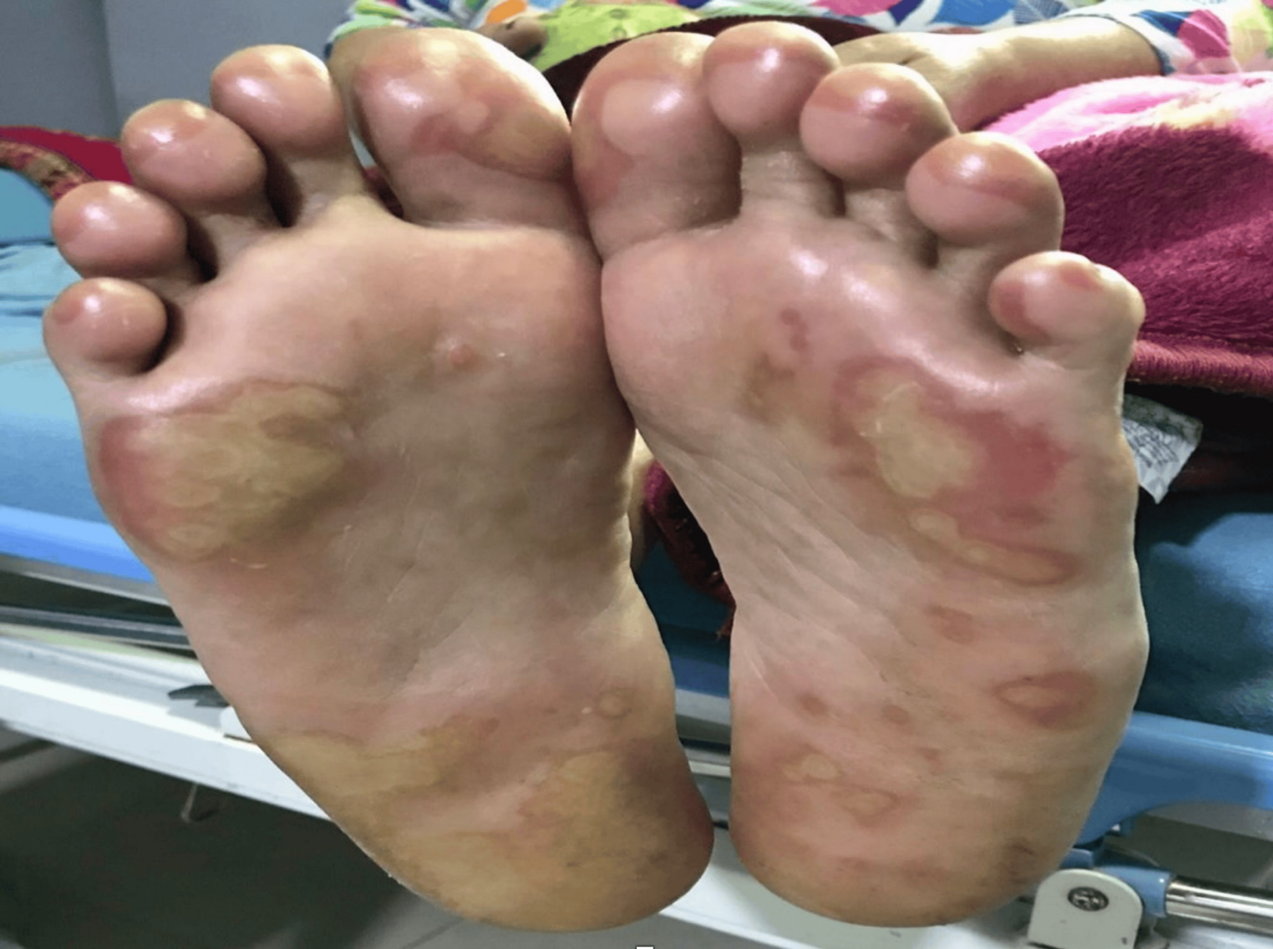
Presence of multiple bilateral erythematous plaques on the soles of the feet

**Figure 2:**
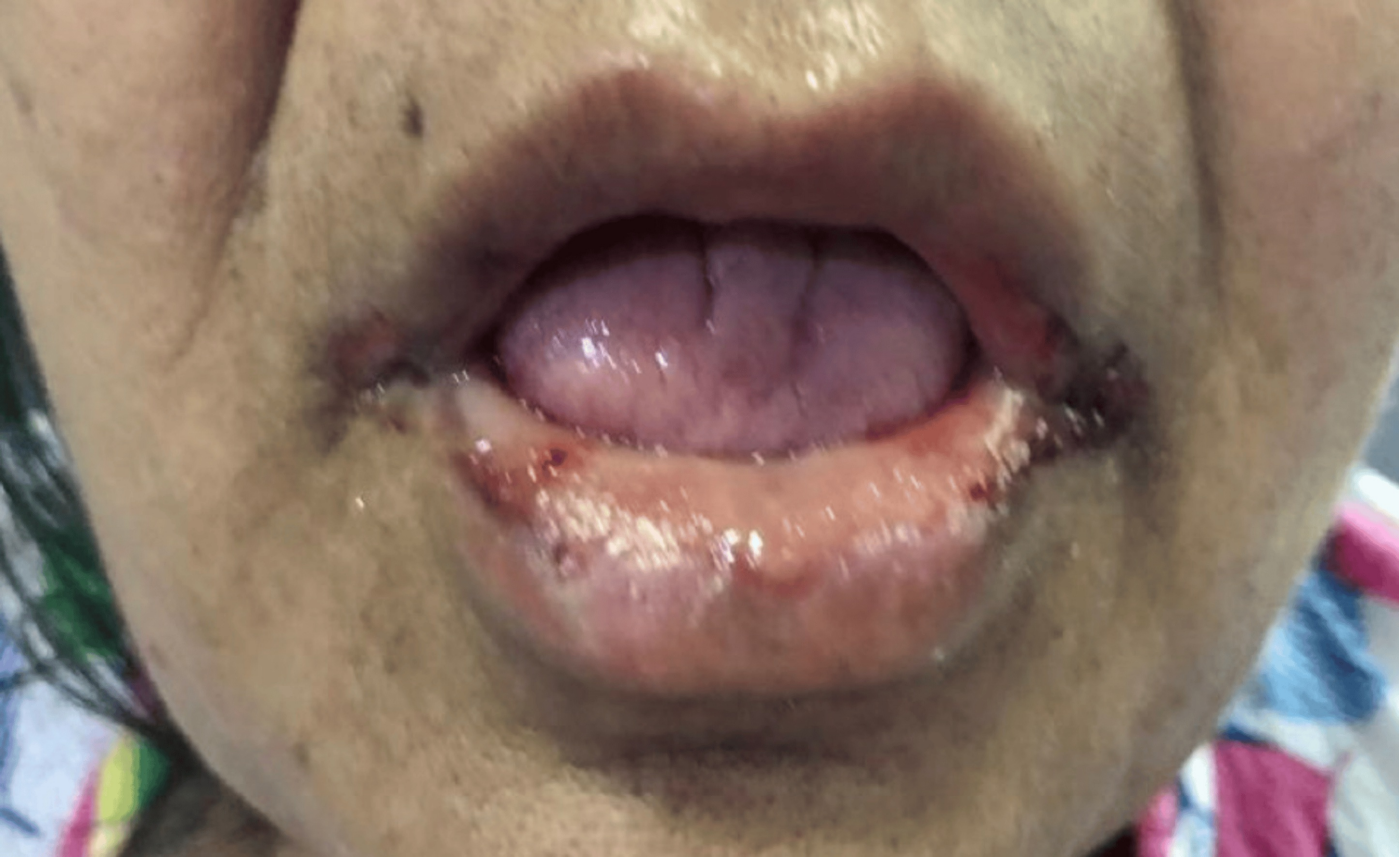
Erosion and haemorrhagic crusting over the lower lip and angles of the mouth

Upon examination, we noted multiple erythematous ulcerated plaques over the skin of the palms, soles of the feet (Figure 1), and lower abdomen. There were ulcers on her lips, and the angle of her mouth was indicative of mucositis (Figure 2), as well as an erythematous ulcer extending throughout her soft palate to the posterior pharyngeal wall. Her vital signs were indicative of fever, with a documented temperature of 99.9°F, and she appeared fatigued and ill-looking.

Investigations

Laboratory tests showed anaemia (Hb: 9.5 g/dL), leukopenia (WBC: 1800/cu.mm), with neutrophilia (neutrophils 68% and absolute neutrophil count (ANC) 1224). Her platelet count was 150,000/cu.mm. Peripheral blood smear also confirmed pancytopenia, and the stool occult blood test was positive, indicative of possible bleeding in the gastrointestinal tract. Her erythrocyte sedimentation rate and C-reactive protein were both elevated at 55 mm/hour and 96 mg/dL, respectively. Mild impairment in her liver function tests was noted, with aspartate aminotransferase (AST) at 14.7 U/L, alanine aminotransferase (ALT) at 57.44 U/L, and alkaline phosphatase (ALP) at 64.15 IU/L. Her bone marrow function was poor, as indicated by a low reticulocyte count of 0.28% (normal range: 0.5%-2.5%). Additionally, her rheumatoid factor was mildly positive at 8 IU/L. The chest X-ray was unremarkable, and a biopsy from the skin lesions was advised but refused by the patient.

Diagnosis and management

The acute onset of symptoms, combined with the patient’s medication history, physical examination, and laboratory test results, led to the suspicion of MTX toxicity. Subsequently, she was admitted to the medicine ward for further evaluation and management. On the third day of her hospitalisation, her condition worsened, with a temperature rise (103°F) and an additional drop in blood counts, with Hb at 8 g/dL, WBC 500/cu.mm, ANC 175, and platelets 45,000/cu.mm. We diagnosed her with neutropenic fever and myelosuppression owing to MTX toxicity. She was started on intravenous antibiotics (meropenem and amikacin), oral folic acid, folinic acid (leucovorin) to counteract MTX toxicity, and synthetic colony-stimulating factor (filgrastim) to stimulate WBC production, along with blood transfusion as part of her treatment plan.

Her condition improved following treatment, and by the seventh day of admission, she was afebrile, and oral erosions and palmoplantar targetoid skin lesions were healing. There was an improvement in blood counts (Hb 9.9 g/dL, WBC 8,400/cu.mm, ANC 6,216, and platelets 125,000/cu.mm), owing to which leucovorin and filgrastim were discontinued. A dermatology consultation was sought, and topical antibiotics, folic acid mouthwash, and gargle were initiated based on their advice. The patient was discharged with appropriate medications, and follow-up was scheduled a week after discharge.

## Discussion

MTX, a drug with anti-folate properties, exhibits both cytotoxic and immunosuppressive effects. These characteristics make it a valuable therapeutic tool in managing specific cancers, psoriasis, and RA. The liver metabolises the drug, and the kidneys primarily eliminate it. Pharmacokinetic data reveal that over 90% of the medication is cleared from the body within the first 24 hours of administration [3]. MTX is widely used in the treatment of various autoimmune rheumatic disorders, such as RA, psoriatic arthritis, and lymphomas [4]. While nausea, vomiting, and diarrhoea are frequent side effects of MTX therapy, folic acid supplementation can help lessen their impact. Studies suggest several factors may increase a patient’s susceptibility to MTX toxicity, including higher BMI, older age, elevated mean corpuscular volume, reduced creatinine clearance, a history of digestive issues from prior MTX use, low albumin levels, and being female [5].

Fortunately, the risk of MTX causing pancytopenia is rare, occurring in only about 1.4% of cases [6]. Bone marrow toxicity from MTX can occur in two main ways. It can be a dose-dependent cumulative effect that develops over years of use, or it can be related to genetic variations in the MTHFR gene [7]. Specifically, polymorphisms in the A1298C and C677T variants can lead to a decrease in MTHFR activity, potentially increasing the risk of MTX toxicity [8].

A retrospective analysis of 70 patients (1980-1995) explored factors contributing to MTX toxicity. All patients experienced severe complications due to pancytopenia. Interestingly, the average cumulative MTX dose was relatively low (675 mg). The study revealed several potential risk factors. A significant portion (58%) had pre-existing kidney problems, and many (40%) were taking slow-acting antirheumatic drugs (SAARDs) before starting MTX. Polypharmacy was also common, with 12 patients on over five additional medications. Underlying infections (51%) and low albumin levels (24%) were further contributing factors. Notably, even very low MTX doses (10 mg) proved fatal in two patients with these risk factors. This study highlights the importance of careful patient evaluation before initiating MTX therapy. Impaired renal function, polypharmacy, infections, and low albumin levels may significantly increase the risk of severe pancytopenia [9].

Any suspicion of MTX toxicity warrants immediate discontinuation of the medication and initiation of leucovorin therapy. This intervention should not be delayed under any circumstances [2]. Persistent nausea and vomiting from MTX can be treated with various antiemetics. Intravenous fluids and electrolyte management are crucial. Consider leucovorin rescue for suspected toxicity. In severe cases, granulocyte colony-stimulating factors, blood transfusions, and supportive care for bleeding, infections, and organ function are necessary. Monitor closely, and treat respiratory symptoms and skin issues.

This case underscores the need for careful consideration when using MTX in elderly patients. To ensure medication safety, clinicians should provide clear and specific instructions. This includes details like the exact number and strength of tablets, the intended dosing time, and separate, designated days for MTX and folic acid administration [10].

## Conclusions

In conclusion, this case highlights an atypical presentation of severe MTX toxicity in an elderly patient experiencing extensive bone marrow suppression. While uncommon, recognising potential risk factors for such reactions is crucial. Future research, potentially through clinical trials, is needed to identify patients more susceptible to idiosyncratic reactions and genetic variations that could lead to life-threatening MTX toxicity.

## References

[1] Lopez-Olivo MA, Siddhanamatha HR, Shea B, Tugwell P, Wells GA, Suarez-Almazor ME (2014). Methotrexate for treating rheumatoid arthritis. Cochrane Database Syst Rev.

[2] Weidmann A, Foulkes AC, Kirkham N, Reynolds NJ (2014). Methotrexate toxicity during treatment of chronic plaque psoriasis: a case report and review of the literature. Dermatol Ther (Heidelb).

[3] Henderson ES, Adamson RH, Oliverio VT (1965). The metabolic fate of tritiated methotrexate. II. Absorption and excretion in man. Cancer Res.

[4] Kumari P, Jain S, Sonakusale P (2021). Idiosyncratic reaction to single 7.5mg dose of methotrexate: mishappening by chance. CHRISMED J Health Res.

[5] Lim AY, Gaffney K, Scott DG (2005). Methotrexate-induced pancytopenia: serious and under-reported? Our experience of 25 cases in 5 years. Rheumatology (Oxford).

[6] Cansu DÜ, Teke HÜ, Bodakçi E, Korkmaz C (2018). How should we manage low-dose methotrexate-induced pancytopenia in patients with rheumatoid arthritis?. Clin Rheumatol.

[7] Ajmani S, Preet Singh Y, Prasad S (2017). Methotrexate-induced pancytopenia: a case series of 46 patients. Int J Rheum Dis.

[8] Ulrich CM, Yasui Y, Storb R (2001). Pharmacogenetics of methotrexate: toxicity among marrow transplantation patients varies with the methylenetetrahydrofolate reductase C677T polymorphism. Blood.

[9] Gutierrez-Ureña S, Molina JF, García CO, Cuéllar ML, Espinoza LR (1996). Pancytopenia secondary to methotrexate therapy in rheumatoid arthritis. Arthritis Rheum.

[10] Amissah-Arthur MB, Baah W (2020). Methotrexate-induced pancytopenia and mucositis caused by medication error. Ghana Med J.

